# Microscopic analysis of the microbiota of three commercial Phytoseiidae species (Acari: Mesostigmata)

**DOI:** 10.1007/s10493-020-00520-3

**Published:** 2020-07-07

**Authors:** Jason C. Sumner-Kalkun, Ian Baxter, M. Alejandra Perotti

**Affiliations:** 1 SASA, Roddinglaw Road, Edinburgh, EH12 9FJ UK; 2 Certis Europe BV, Stadsplateau 16, 3521 AZ Utrecht, The Netherlands; 3 Ecology and Evolutionary Biology Section, School of Biological Sciences, University of Reading, Reading, RG6 6AS UK

**Keywords:** Phytoseiidae, Mass rearing, Microscopy, Microbiome, Acari, FISH

## Abstract

Microbes associated with the external and internal anatomy of three commercially available predatory mite species—*Phytoseiulus persimilis*, *Typhlodromips* (= *Amblyseius*) *swirskii*, and *Neoseiulus* (= *Amblyseius*) *cucumeris*—were examined using light microscopy, confocal laser scanning microscopy and fluorescence in-situ hybridization (FISH). Four microbe morphotypes were observed on external body regions. These included three microfungi-like organisms (named T1, T2 and T3) and rod-shaped bacteria (T4). Morphotypes showed unique distributions on the external body regions and certain microbes were found only on one host species. Microfungi-like T1 were present in all three species whereas T2 and T3 were present in only *P. persimilis* and *T. swirskii*, respectively. T1 and T2 microbes were most abundant on the ventral structures of the idiosoma and legs, most frequently associated with coxae, coxal folds, ventrianal shields and epigynal shields. T3 microbes were most abundant on legs and dorsal idiosoma. T4 microbes were less abundant and were attached to epigynal shields of *N. cucumeris* and *T. swirskii.* Significant differences in distribution between batches suggest temporal fluctuations in the microbiota of phytoseiids in mass-reared systems. FISH showed bacteria within the alimentary tract, in Malpighian tubules and anal atria. These may aid absorption of excretory products or maintaining gut physiology. We suggest a mechanism by which microbes may be transmitted to offspring and throughout populations. This study aims to improve our knowledge of this poorly understood area and highlights the necessity of understanding the microbiota of Acari.

## Introduction

The mite family Phytoseiidae (Acari: Mesostigmata) contains 2789 described species (Demite et al. 2014). They are generally found on aerial parts of plants and sometimes in soil. Many species feed on micro-invertebrates such as mites, insects and nematodes, whereas others are fungal feeders or feed on pollen and exudates of plants (Gerson et al. 2003). The Phytoseiidae receive interest due to their importance as biological control agents of pest mites, whiteflies, scale insects and thrips (Gerson et al. 2003; Hoy 2011). The three species of phytoseiids examined in this study—*Phytoseiulus persimilis* Athias-Henriot, *Neoseiulus* (= *Amblyseius*) *cucumeris* (Oudemans), and *Typhlodromips* (= *Amblyseius*) *swirskii* (Athias-Henriot)—are all mass reared by commercial companies worldwide. *Phytoseiulus persimilis* is a specialised predator of spider mites (van Lenteren and Woets 1988; McMurtry and Croft 1997; Zhang 2003), whereas *N. cucumeris* and *T. swirskii* are used mainly to control thrips (Gillespie 1989; Grafton-Cardwell et al. 1999; De Courcy Williams 2001; Shipp and Wang 2003; Messelink et al. 2005; van Houten et al. 2005; Van Driesche et al. 2006; Arthurs et al. 2009; Doğramaci et al. 2011). *Neoseiulus cucumeris* has also been used in the control of pest mites such as *Polyphagotarsonematus latus* (Banks) (Weintraub et al. 2003) and *Phytonemus pallidus* (Banks) (Easterbrook et al. 2001). *Typhlodromips swirskii* is used for controlling the widely distributed tobacco whitefly, *Bemisia tabaci* (Gennadius) (Hoogerbrugge et al. 2005; Fouly et al. 2011) and, in some cases, the greenhouse whitefly *Trialeurodes vaporariorum* Westwood (Messelink et al. 2008)*.* Furthermore *T. swirskii* and *N. cucumeris* have been used to effectively spread fungal spores of *Beauveria bassiana* to improve the biological control of *Diaphorina citri*, the citrus psyllid (Zhang et al. 2015). Despite their importance as biological control agents, little is known of the intrinsic Phytoseiidae microbiota or how their morphology interacts with microbes within the environment or intrinsic microbiota. Microbes with pathogenic effects have been relatively well described (Bjørnson et al. 2000; van der Geest et al. 2000; Pukall et al. 2006; Bjørnson 2008; Schütte et al. 2008; Bruin and van der Geest 2009); however, little is known about other microbial partners regularly associated with asymptomatic phytoseiids.

In terms of microbiota, the most comprehensively studied phytoseiids are *Metaseiulus occidentalis* Nesbitt (Hoy and Jeyaprakash 2005) and *N. cucumeris* (Pekas et al. 2017). In *M. occidentalis* bacteria were isolated in tissues of the digestive tract, reproductive tract and in eggs (Hess and Hoy 1982; Jeyaprakash and Hoy 2004; Hoy and Jeyaprakash 2005). For *N. cucumeris* Next Generation Sequence (NGS) approaches to microbiota characterization were used to explore the diversity of different populations of this predator mite and its factitious prey *Tyrophagus putrescentiae* (Schrank) (Pekas et al. 2017). Studies comparing phytoseiid microbiota to their prey microbiota showed that predator and prey share core bacterial species (Hoy and Jeyaprakash 2005; Pekas et al. 2017). However, the intrinsic bacterial microbiota of *N. cucumeris* and its factitious prey *T. putrescentiae* were significantly different, and the presence of predatory mites in prey populations caused significant shifts in the microbiota of *T. putrescentiae* (Pekas et al. 2017). Previous work on the phytoseiid microbiota showed they are dynamic systems affected by the environment and by the microbiota of other species. Microbial communities may also play an important role in health, disease and physiology of these mites.

Compared to the large and growing body of work considering the impact of insect microbiota, the roles of microbiota in the Acari have not received the attention they deserve. Works by Reichenow (1922) and Piekarski (1935) (reviewed by Buchner 1965) pioneered early studies into the microbiota of the Mesostigmata (= Gamasida). These manuscripts described the presence of potentially symbiotic bacteria associated with the gut of some species of blood-sucking mites. A few isolated cases of mite and tick species of economic importance have gathered some attention particularly in relation to eukaryotic symbiont microbes (Perotti and Braig 2011). The small amount of existing studies on the microbial associates of the Acari suggest that the mite microbiota fulfils a number of different roles, for example: improving nutrition and digestion in oribatids, astigmatids, tarsonemids and uropodid mites (Moser 1985; Smrž and Trelová 1995; Klepzig et al. 2001; Roets et al. 2007; Hubert et al. 2011, 2015b, 2016a); aiding reproduction in ticks and macrochelids (Feldman-Muhsam 1991; Perotti and Braig 2011); and conferring resistance to acaricides (Yoon et al. 2010). In addition to gut and internal microbiota, mites also possess an external microbiota with which they interact. Some *Tricouropoda* and Tarsonemidae species house fungal spores and bacteria in specialised areas of the ventral body surface called sporothecae (Moser 1985; Roets et al. 2007, 2011). These adaptations have arisen due to the necessity for the mite to cultivate symbiotic interactions with these microbes and retain them as part of their core microbiota. The Tarsonemidae also possess cerotegument, a waxy substance which coats the body of these mites. The cerotegument is often found with fungi, lichen and bacteria attached and is suggested as a mechanism for these mites to disseminate beneficial microorganisms and plant pathogens (Rezende et al. 2015, 2017).

It is essential to identify the microbiota of phytoseiids and the potential effects they may have on the health and performance of mites in mass rearing and biological control applications. Producers aim to supply the fittest predatory mites possible and indications that diseases such as non-responding syndrome (Pukall et al. 2006; Schütte et al. 2008) could be causing deleterious effects on mite fecundity and longevity, add additional relevance this study. This work uses microscopy techniques to morphologically examine the external (surface) microbiota and compares their localisation and distribution on the body and digestive tract of *P. persimilis*, *T. swirskii* and *N. cucumeris*. Prokaryotes and eukaryotes were observed and where possible targeted using light microscopy, confocal laser microscopy and fluorescence in-situ hybridization (FISH). Observations of microbiota were also carried out within their internal anatomy, specifically in digestive tract. Specific body regions were examined to understand the structures with which microbes commonly associate.

## Materials and methods

### Mite populations

*Phytoseiulus persimilis*, *T. swirskii* and *N. cucumeris* originated from mass-reared colonies supplied by Certis Europe. Specimens of mites were taken at various time points to assess changes in microbial distributions over time. All specimens examined were females. These specimens came from populations from different batches: batch 1 from October 2011 and batch 2 from April 2013. The numbers of mites studied from each batch were as follows: *P. persimilis* 37 and 28, *N. cucumeris* 33 and 29, and *T. swirskii* 20 and 31, from batch 1 and batch 2, respectively.

### Observations of external microbiota of phytoseiids

Comparisons between microbes on the integument of *P. persimilis*, *N. cucumeris* and *T. swirskii* were performed using light microscopy and staining techniques. Frequency, size, morphology and exact location of microorganisms were recorded for each individual specimen. Microbes were classified during preliminary examinations of Phytoseiidae mites and four types were observed. These were classified using their gross morphology, general shape, size, dye affinity and any other unique characteristics.

A Gram’s stain technique was adapted from Gerhardt et al. (1981) to improve contrast between microbes and host. Mites were initially fixed in > 90% ethanol. Specimens were rehydrated in sterile H_2_0 for at least 30 min. Mites were transferred using a looped micro-wire into watch glasses containing reagents in the following order: crystal violet for 60 s; H_2_0 to rinse; Gram’s iodine for 60 s; H_2_0 to rinse; 75% ethanol for 3 s; H_2_0 to rinse; safranin for 60 s and H_2_0 to rinse. Mites were mounted on slides with Hoyer’s mounting media and examined using a Nikon Optiphot phase contrast light microscope up to × 1500 magnification. Frequency, size, morphology and exact location of microorganisms were recorded for each mite examined. For data analyses, microbe locations were categorised as one of the following: (1) dorsal idiosoma, (2) ventral idiosoma, (3) legs or (4) gnathosoma. Pictures of each individual mite were taken and the surface area of each of the above categories was measured using ImageJ software v.1.2 (Schneider et al. 2012).

### Observations of internal microbiota using FISH

Fluorescence in-situ hybridization (FISH) was used to localise bacteria within the digestive tract of the three Phytoseiidae species. Other mites and insects have been shown to possess bacteria within their gut and this study aimed to find parallels within the Phytoseiidae and examine differences between phytoseiid species. The 16S rRNA gene region was targeted using oligonucleotide probes that cover most lineages of bacteria: EUB338 (Amann et al. 1990), EUB338-II and EUB338-III (Daims et al. 1999). Probes were labelled with Cy5 fluorophore (em649/ex666) enabling subsequent visualisation and verification of successful hybridization. Hybridization was performed in hybridization buffer (HB) (Perotti et al. 2007). Mites were placed in 200 µl HB for at least 30 min. HB was then refreshed to 200 µl and 1 µl of each probe was added to the tube (0.5 pmol final concentration). Samples were incubated at 45 °C for 4–5 h to allow probe hybridization within the lumen of the digestive tract. After incubation, mites were washed again in fresh HB, mounted in 50% glycerol (and sterile distilled water) and examined using a Zeiss LSM710 confocal laser-scanning microscope. Negative control mites were prepared as above but with no probes added to ensure fluorescence was not an artefact of the preparation process or natural epifluorescence.

### Statistical analyses of external microbe distribution

Counts of microbes and areas of colonization were recorded for each type of microbe on individual mites. For modelling distribution, microbe locations were categorised into four body regions: legs, gnathosoma, dorsal idiosoma and ventral idiosoma. Estimates of the total surface areas of these anatomies were measured for each individual mite using Image-J software v.1.2 (Schneider et al. 2012). Regression of counts was analysed using best fit models comparing generalised linear models (GLMs) to examine the effects of different predictor variables on microbe distribution. Explanatory variables used were body region, batch (comparing differences in microbe distributions among time points) and the batch-body region interaction. Models were fitted to a quasi-Poisson distribution family to account for over-dispersion. Different anatomies were weighted by incorporating the log of their surface area into the models as an offset. Minimum adequate models were found by removing non-significant terms. Goodness of fit for GLMs was compared using analysis of variance (ANOVA) with anatomy and batch as explanatory variables; analysis of deviance was performed using F-tests. Multiple pair-wise comparisons were performed using Tukey’s test. P-values were adjusted using Bonferroni correction to account for multiple tests on the same sample set. Statistical analyses of microbe types were performed on light microscopy observation and not on observations from FISH. All statistical analyses were performed using R (R Core Team 2017).

## Results

### Microbes types and their distribution on the phytoseiid host integument

**Fig. 1:**
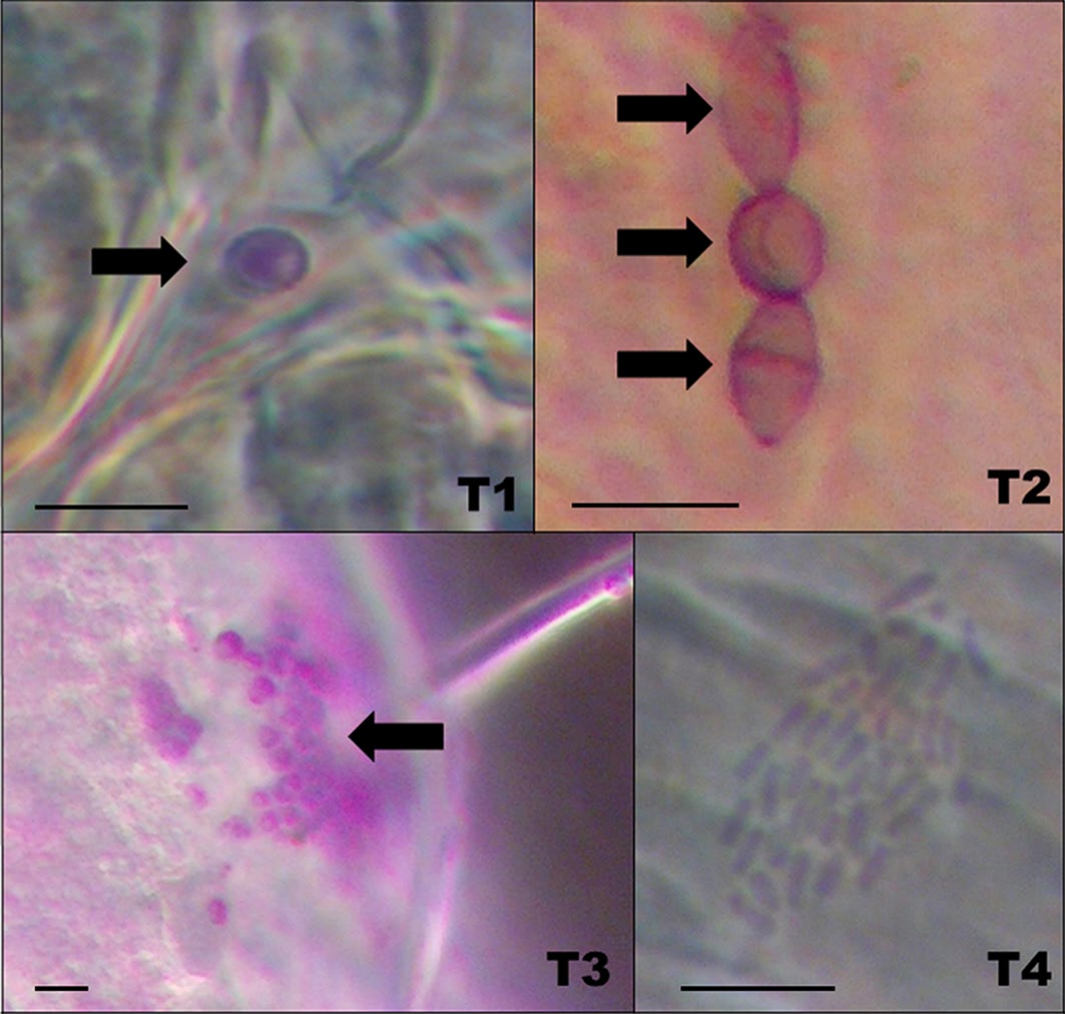
Microorganisms commonly found on the integument of three phytoseiid species: *Neoseiulus cucumeris*, *Phytoseiulus persimilis*, and *Typhlodromips swirskii*. T1 are microfungi-like microbes found in all species, with a rounded cup shape with small protrusions on the surface of the cell (mean [± SD] length = 2.64 ± 0.38 µm, width = 2.54 ± 0.35 µm). T2 are yeast-like microbes only found in *P. persimilis.* They had an egg-shaped cell often with darker sections at the tip of the cell (length = 4.57 ± 1.12 µm, width = 3.25 ± 0.71 µm). T3 are smaller globular cells that commonly formed aggregations on the integument of *T. swirksii* (length = 3.1 ± 0.51 µm, width = 2.98 ± 0.48 µm); based on their size these are suggested to be microfungi. T4 microbes are small bacterial rod-shaped cells (length = 1.13 ± 0.07 µm) and were found on the integument of *N. cucumeris* and *T. swirskii.* Scale bars represent 5 µm

Microbes present on the integument of the mites were primarily divided into four morphotypes by describing shape, size, dye affinity or other characteristics; these are all represented in Fig. 1, which summarises the various types.

**Fig. 2:**
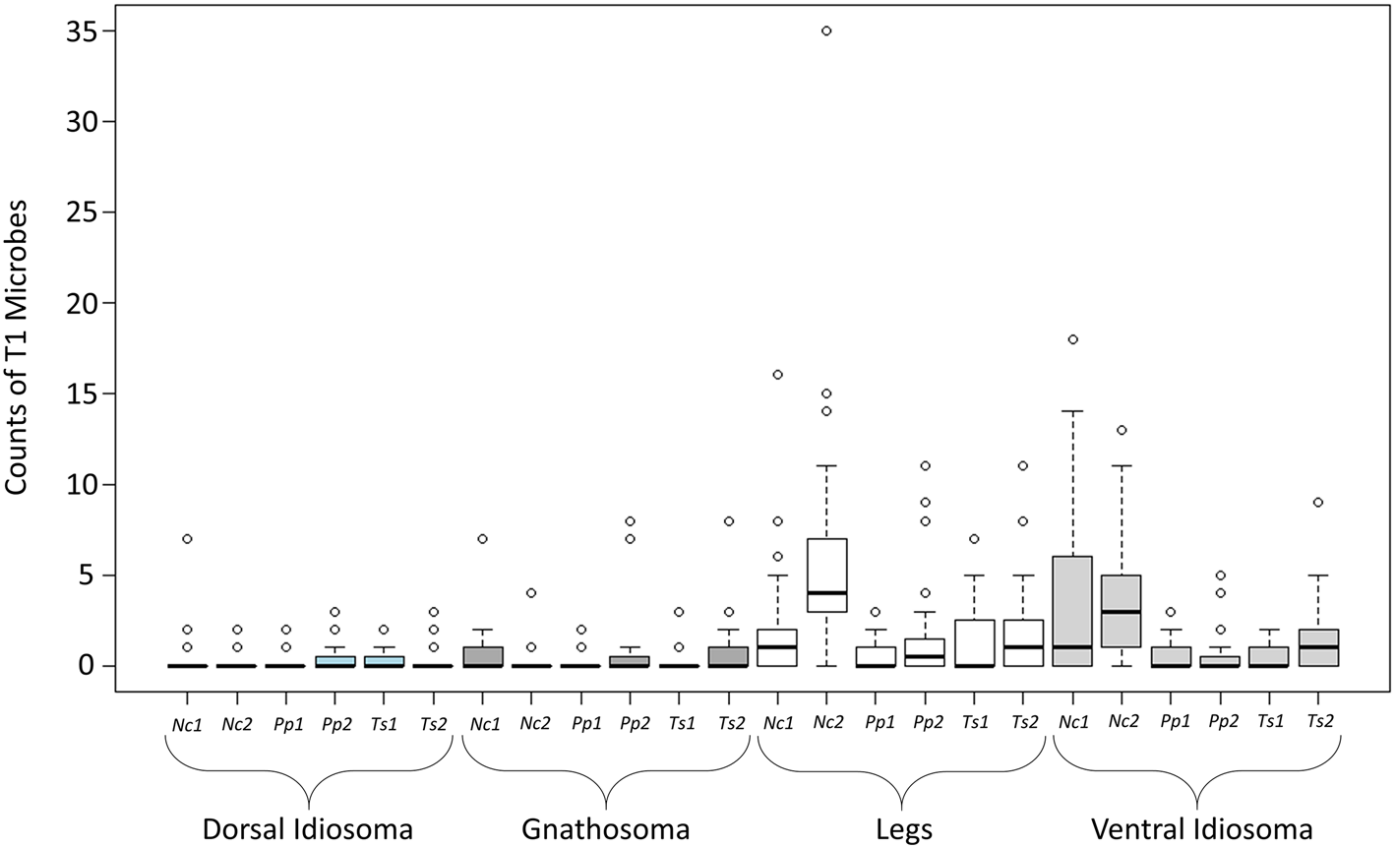
Box plots of counts of type 1 microbes (T1) on all mite species examined. X-axis shows mite species (*Nc* = *Neoseiulus cucumeris*, *Pp* = *Phytoseiulus persimilis*, *Ts* = *Typhlodromips swirskii*), batch (1 = winter, 2 = spring) and body region. Counts were highest for all species on legs and ventral idiosomas. Box plots show median (thick line within) and the lower and upper quartiles below and above, respectively. Whiskers show the minimum and maximum number of counts

**Fig. 3:**
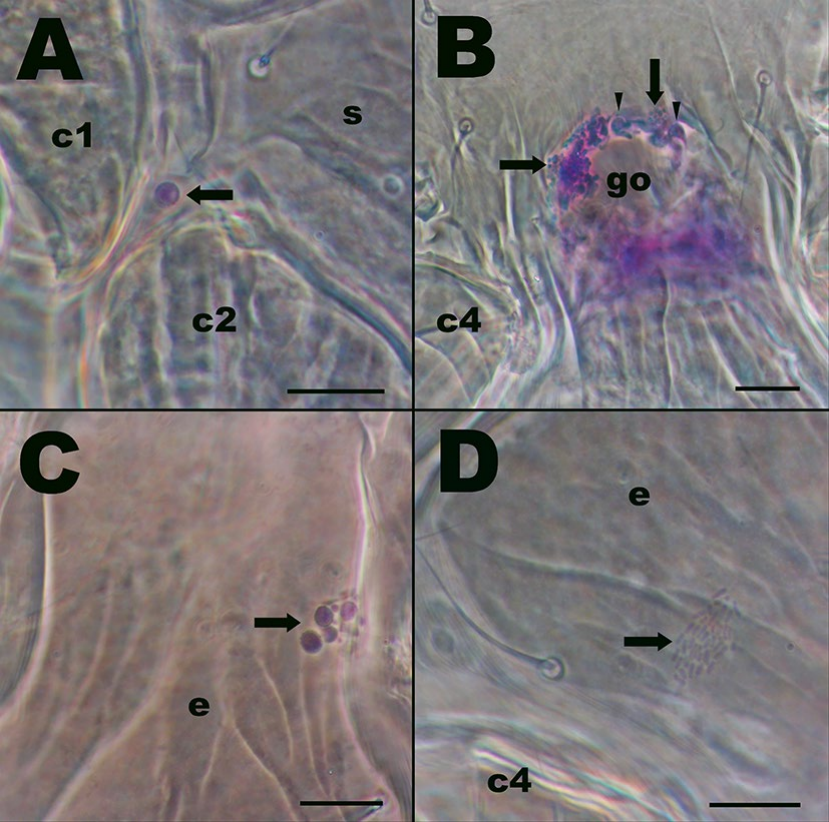
The T1 and T4 microbes associated with *Neoseiulus cucumeris*, adult female venters. **a** T1 microbes between right coxae 1 (c1) and 2 (c2). **b** T1 microbes around the genital opening (go) between the 4th coxal pair (c4); arrows show smaller spores and arrow heads show larger spores. **c** T1 microbes on margin of the epigynal shield (e). **d** Rod-shaped bacteria-like organisms attached to epigynal shield (e) near left coxa 4 (c4). Scale bars represent 20 µm

Type 1 microorganisms (T1) were characterised by a rounded cup shape, with minute protuberances across the entire surface. T1 were present in each mite species examined and were on average 2.64 (± 0.38 SD) µm in diameter (Fig. 1, T1). Based on their size and shape, they are possibly fungal conidia. In each mite species T1 microbes were found to infect > 60% of the population and were the most frequently occurring microbe of the four types described here (Table 1). In all mite species, T1 microbes were most abundant on the legs and ventral idiosoma (Fig. 2). Microbes showed specific sites of colonisation on these body regions and were most abundant ventrally between coxae (Fig. 3a), in the folds at the base of coxae, on epigynal shields (Fig. 3b), and on the coxae, trochanters and tarsi of the legs.

**Table 1:** Infection (% mites) of *Typhlodromips swirskii*, *Neoseiulus cucumeris* and *Phytoseiulus persimilis*, and average number of microbes per mite for all microbes observed in two batches

Species	Microbe type	Mean no. microbes		% mites infected	
		Batch 1	Batch 2	Batch 1	Batch 2
*Typhlodromips swirskii*	Type 1	2.4	4.55	75	96.8
	Type2	0	0	0	0
	Type3	80.80	0	68.4	0
	Rod-shaped bacteria	0	1.32	0	3.2
*Neoseiulus cucumeris*	Type 1	6.06	10.27	85.3	100
	Type2	0	0	0	0
	Type3	0	0	0	0
	Rod-shaped bacteria	2.80	0	9.09	0
*Phytoseiulus persimilis*	Type 1	1.54	3.61	67.6	78.6
	Type2	11.49	2.71	97.2	50
	Type3	0	0	0	0
	Rod-shaped bacteria	0	0	0	0

Sample sizes: *T. swirskii*, batch 1 (n = 20), batch 2 (n = 31); *N. cucumeris*, batch 1 (n = 33), batch 2 (n = 29); *P. persimilis*, batch 1 (n = 37), batch 2 (n = 28)

Specimens of *N. cucumeris* had high numbers of T1 microbes around the genital opening (Fig. 3c). Differences in distributions of T1 between batches were only found in *P. persimilis* (ANOVA: *F*_1,255_ = 10.225, *P* = 0.0047), whereas in *T. swirskii* and *N. cucumeris* batch was a non-significant factor (Table 2). However, phytoseiid body region was always a significant factor affecting distribution of T1 microbes: *T. swirskii* (*F*_3,199_ = 4.021, *P* < 0.05), *P. persimilis* (*F*_2,255_ = 4.8684, *P* < 0.01) and *N. cucumeris* (*F*_3,243_ = 14.784, *P* < 0.001) (Table 2). In *P. persimilis* overall, T1 microbes were more frequent on legs compared to gnathosoma, but were also more frequent on the gnathosoma than they were on the dorsal idiosoma. On *T. swirskii*, T1 microbes were more frequent on both the ventral idisoma and gnathosoma compared to the dorsal idiosoma. And in *N. cucumeris* T1 microbes were more frequent on the ventral idiosoma compared to the gnathosoma, and overall ventral body region had higher frequencies of T1 microbes compared to dorsal idiosoma (Table 2).

**Table 2:** GLM comparisons with ANOVA to find minimum adequate models describing factors affecting the distribution of various types of microbes on three mass-reared Phytoseiidae species

Species	Microbe type	Explanatory variable	Deviance	F	Pr(> F)
*Typhlodromips swirskii*	Type 1	Body region	228.08	14.784	< 0.001
		Batch	15.78	3.0691	0.243
		Body region*batch	46.69	3.5164	0.05
*Phytoseiulus persimilis*	Type 1	Body region	37.62	4.8684	0.008
		Batch	26.34	10.225	0.005
		Body region*batch	3.07	0.403	1
	Type 2	Body region	270.86	5.0727	0.006
		Batch	178.87	10.049	0.005
		Body region*batch	61.27	1.4943	0.65
*Neoseiulus cucumeris*	Type 1	Body region	31.48	4.021	0.025
		Batch	11.26	4.3162	0.12
		Body region*batch	4.16	0.5341	1
	Type 3	Body region	1850.10	32.02	< 0.0001
		Batch	3192.20	165.74	< 0.0001
		Body region*batch	0.00	0	1

**Fig. 4:**
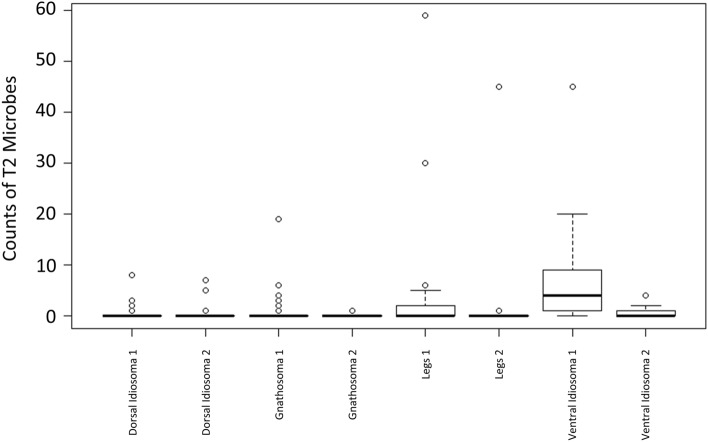
Box plots of counts of type 2 microbes (T2) on *Phytoseiulus persimilis.* X-axis shows body regions and batch (1 = winter, 2 = spring). Counts were highest on legs and ventral idiosoma in winter. See Fig. 2 for an explanation of the boxplot parts

**Fig. 5:**
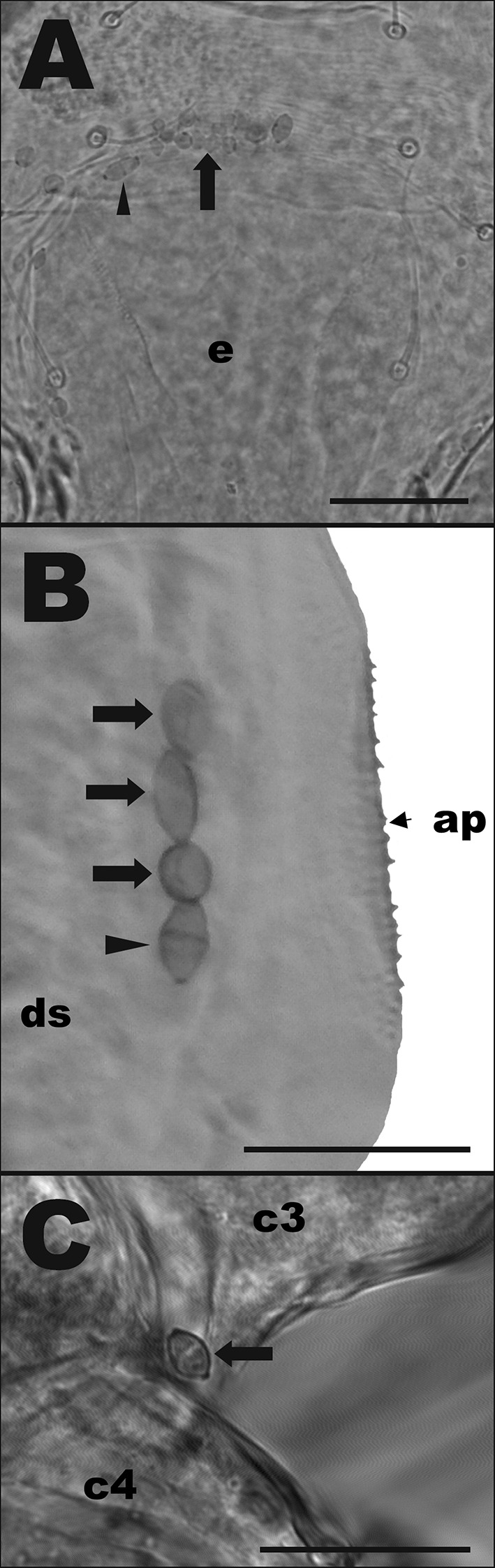
Pictographs of type 2 (T2) microorganisms on *Phytoseiulus persimilis*, adult female venters. **a** Ventral view of T2 microbes associated with the outer margin of the epigynal shield (e). **b** T2 situated at the posterior edge of the dorsal shield (ds); ap, anal plate. **c** T2 located between the bases of coxa 3 (c3) and coxa 4 (c4). Arrow heads point at single cells, arrows at possible dividing cells. Scale bar represents 50 µm (**a**) or 20 µm (**b**, **c**)

Type 2 microorganisms (T2) were unique to *P. persimilis* and were egg-shaped, often with the appearance of budding yeasts and structures that resembled bud scars. T2 microbes are on average 4.57 (± 1.12 SD) µm long (Fig. 1). Based on their size and general characteristics, these are deemed yeast-like organisms. T2 microbes were found in 97.2% of winter specimens but were less prevalent in spring specimens (50%, Table 1), representing a difference in abundance of T2 microbes between batches (*F*_1,255_ = 10.049, *P* < 0.005) (Table 2). These microbes were most abundant on legs and structures of the ventral idiosoma (Fig. 4). They were commonly associated with the outer margins of the sclerotized shield structures of the phytoseiid such as the epigynal shield (Fig. 5a), the dorsal shield (Fig. 5b) and between coxae (Fig. 5c). Distributions of T2 microbes were also different between body regions (*F*_3,255_ = 5.0727, *P* < 0.01) (Table 2), tending to favour the previously mentioned structures. T2 microbes were more frequent on the ventral idiosoma compared to the legs and dorsal idiosoma (Fig. 4).

**Fig. 6:**
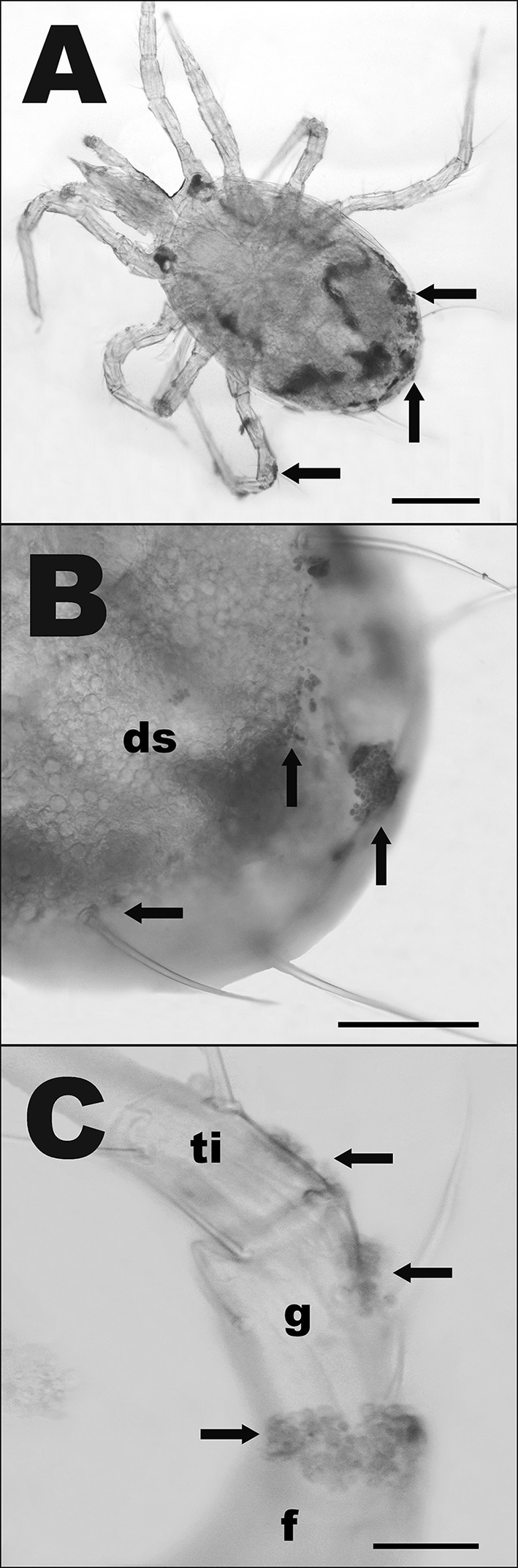
Pictograms of stained type 3 (T3) microorganisms on *Typhlodromips swirskii* adult females. **a** Dorsal view showing the location of T3 microbes on the opisthosoma and legs. **b** T3 on the dorsal shield (ds), opisthosoma and setae. **c** T3 covering the femur (f), genu (g) and tibia (ti) of the left hind leg. Scale bar represents 100 µm (**a**, **b**) or 20 µm (**c**)

Type 3 microorganisms (T3) were only present in *T. swirskii* and were small globular cells that form aggregations covering a large area of the integument of the mite. T3 cells are on average 3.1 µm in diameter (Fig. 1). Due to the size and shape, it is suggested that T3 are also microfungi. Table 1 shows that these microbes were only present in winter specimens (68.4%) and were absent from spring specimens. T3 distribution showed differences between batches (*F*_1,199_ = 165.74, *P* < 0.0001) and between body regions (*F*_3,199_ = 32.02, *P* < 0.0001) (Table 2). Unlike T1 & T2 microbes, T3 microbes were more common on the dorsal idiosoma, colonising large areas of the dorsal opisthosoma, the most posterior section of the mite’s body (Fig. 6a, b). There was also a strong association of T3 microbes with segments of the fourth pair of legs, namely the femurs, genua, tibia and tarsi (Fig. 6c).

Type 4 microorganisms (T4) were found in *N. cucumeris* and *T. swirskii* and are rod-shaped gram-positive bacteria that were observed on the integument of the mite. These were on average 1.13 µm long. These microbes were the least common of all microbes observed (Table 1) and, in the few cases in which they were found, were always associated with the epigynal shield of adult females (Fig. 3d).

### Localization of microbes with FISH and fluorescence microscopy

**Fig. 7:**
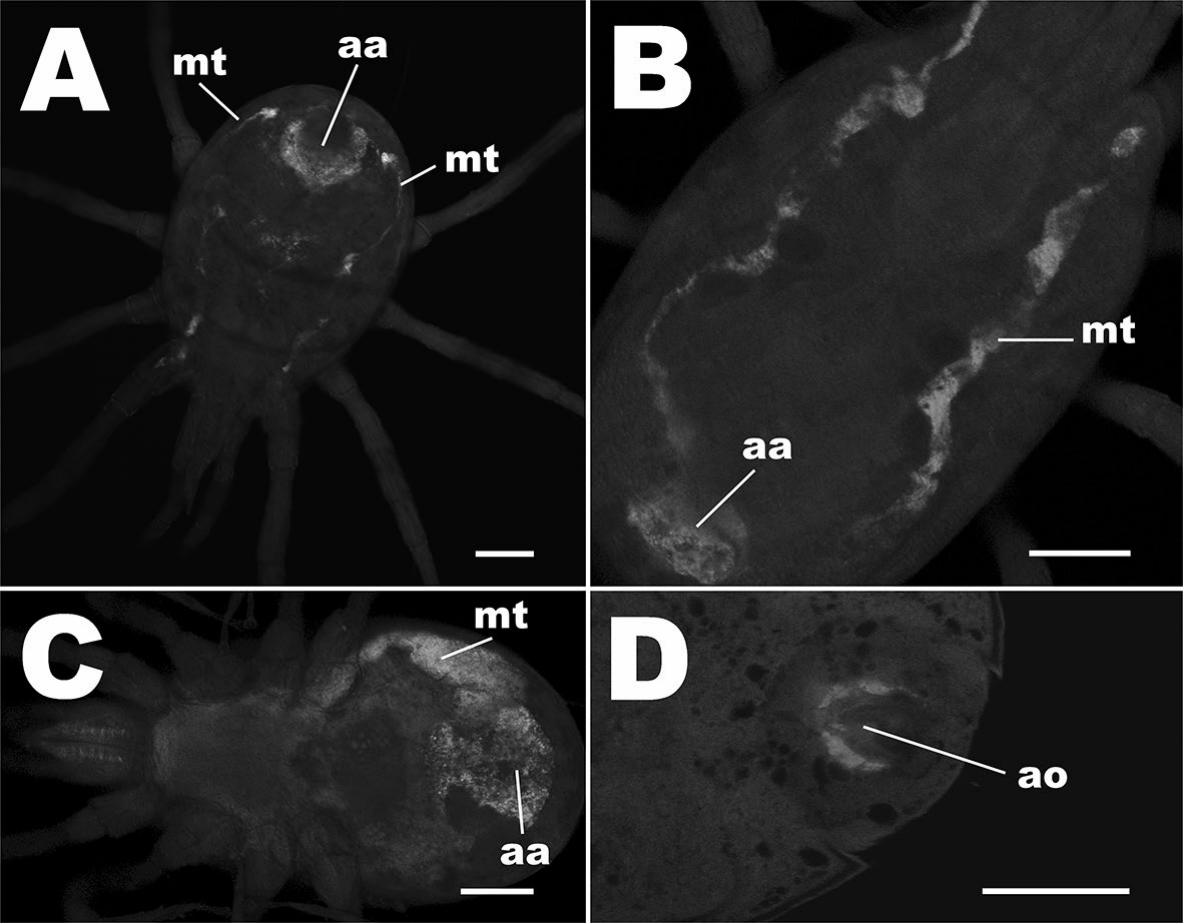
Observations of bacteria using FISH and universal bacteria probes EUB1, 2 and 3, tagged with Cy5 fluorophore. Fluorescent signal is displayed as white/light grey areas, darker grey areas are low-level autofluorescence from the mite integument. See Fig. 8 for control mites without probe showing no signal. Bacteria within the Malpighian tubules (mt) and anal atrium (aa) of **a***Neoseiulus cucumeris*, **b***Phytoseiulus persimilis* and **c***Typhlodromips swirskii*. Malpighian tubules comprise two lobes running parallel along either side of the mite’s opisthosoma, originating from the anal atrium and terminating within the first pair of coxae. **d** Bacteria on the anal opening (ao) of *P. persimilis*. Scale bars represent 50 µm

**Fig. 8:**
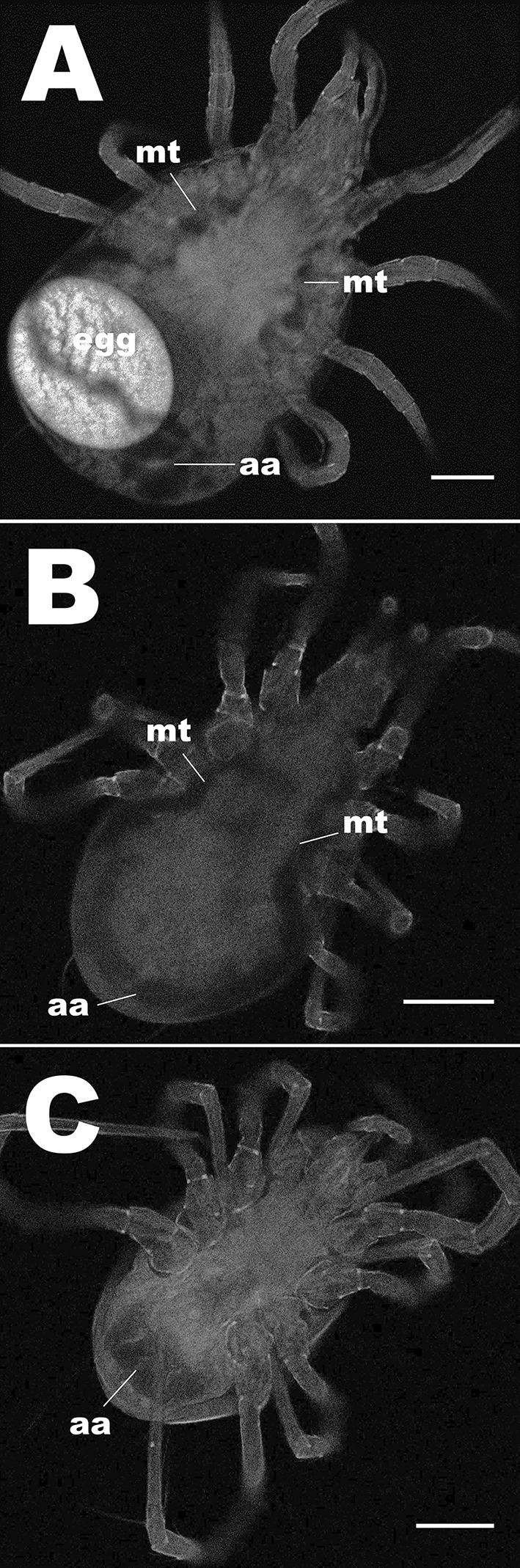
Pictograms of control mites treated with hybridization buffer, but no probe added: **a***Neoseiulus cucumeris*, **b***Typhlodromips swirskii* and **c***Phytoseiulus persimilis*. All mites tested showed no signal in Malpighian tubules (mt) (n = 10 per species; *aa* anal atrium). Scale bars represent 50 µm

**Fig. 9:**
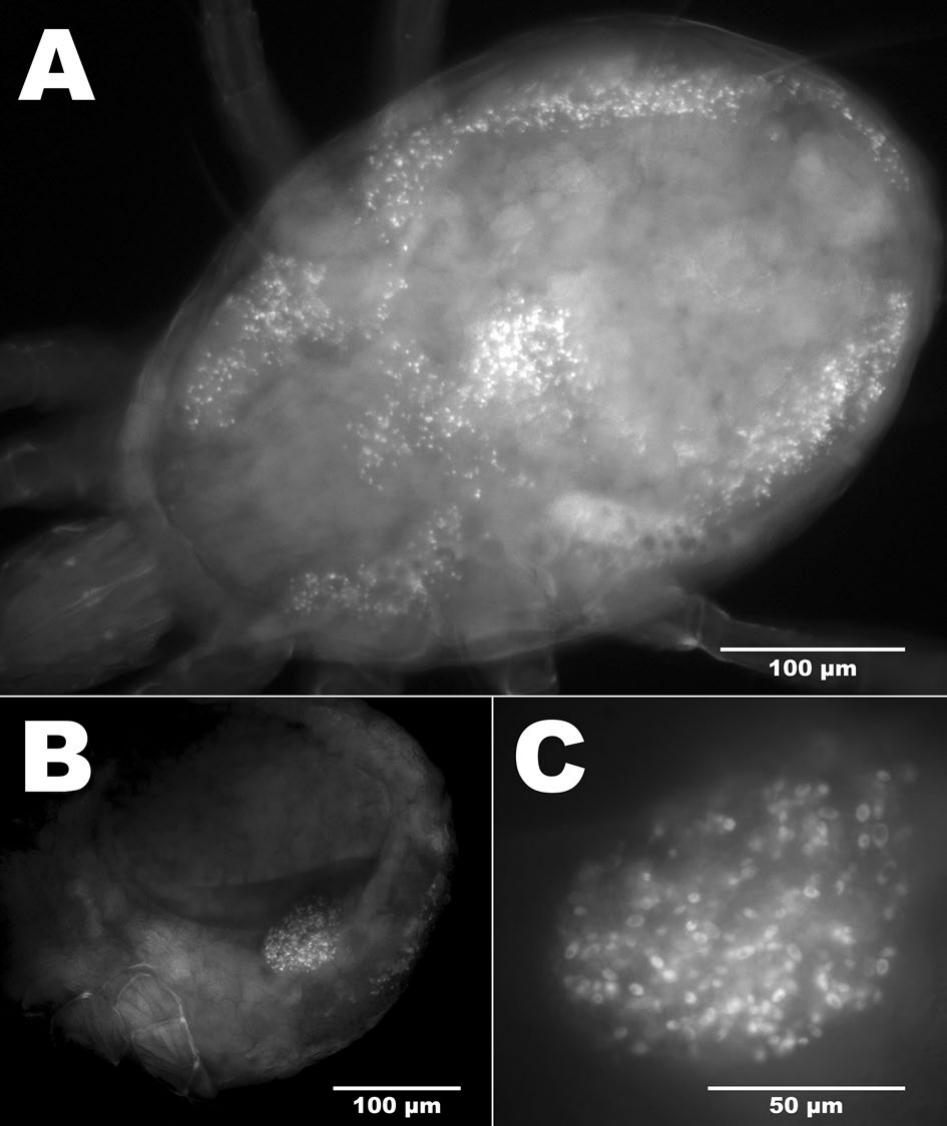
Epifluorescence of fungal spores in the digestive cecae and in reproductive tract of *Typhlodromips swirskii.***a** Whole body view showing spores distributed through each cecal lobe. **b**, **c** Lateral view of spores associated with the egg

**Fig. 10:**
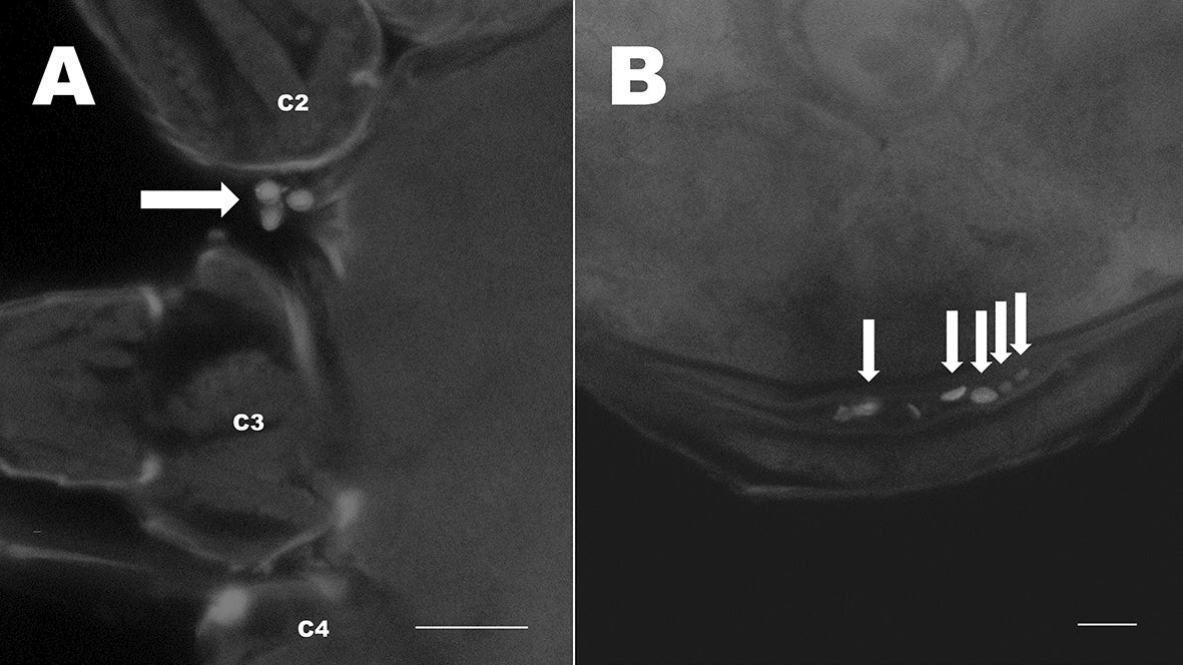
Pictograms of T1 and T2 microbes observed on *Phytoseiulus persimilis* by epifluorescence. **a** Ventral view. Aggregation of T1 microbes situated between the 2nd and 3rd right coxae (c). **b** Posterior half of idiosoma. View of fold between dorsal opisthosoma and ventral opisthosoma harbouring T2 microorganisms observed with epifluorescence. Scale bars represent 10 µm

FISH analysis of the phytoseiids’ digestive tracts showed that each examined species contains bacteria within specific areas. Thin rod-shaped bacteria were situated within the Malpighian tubules and within the anal atria of each mass-reared species tested (Fig. 7a–c) and were on average 0.9 µm long. Additionally in *P. persimilis* bacteria were found covering the circumference of the valves of the anal opening (Fig. 7d). Orthogonal sections of z-stacks showed that in Malpighian tubules and in anal atria, bacterial infection was throughout the lumen represented as solid fluorescence in the orthogonal sections. Bacteria were observed on the surface of the mite on basal segments of the hind legs and around the epigynal shield, again showing that these body regions are important areas for microbial colonisation. Control mites treated with hybridization buffer and no probe did not show signal in the Malpighian tubules (Fig. 8). Although FISH probes were not used to localise fungi on the surfaces and gut lumen of the mites, epifluorescence of fungal cells was used to examine body regions that associated with fungi. Ten percent of *T. swirskii* mites examined with fluorescence microscopy (n = 20) showed fungal cells within the lumen of the digestive tract, with a large number of cells distributed throughout the digestive cecae (Fig. 9a) and fungal cells situated near the developing egg in one female examined (Fig. 9b, c). Using natural epifluorescence microfungi cells with similar shapes and sizes to T1 and T2 organisms were observed on 10% of *N. cucumeris* and 20% of *P. persimilis* (n = 20) (Fig. 10).

## Discussion

### Microbial ecology of mass-reared Phytoseiidae

Overall, microbe types showed unique patterns of distribution and abundances that varied between batches. Body region was always a significant factor affecting the distribution of microbes. The interaction between body region and batch was non-significant in every case suggesting that differences between batches can affect the numbers of microbes present but not the specific body regions with which they associate.

According to this study, mass-reared phytoseiids are hosts to a diversity of microorganisms on the integument and within the digestive tract. These interactions are newly described associations between microbes and phytoseiid hosts. Microbes colonise specific anatomies of their host such as bases of the coxae, epigynal and anal shields, and genital and reproductive openings. Features of some anatomical structures may make them more suited to colonisation by microorganisms. They may provide a suitable substrate on which microbes can attach, provide the microbes with extra nutrient by means of secretions from the mite, or encounter these microbes more often in the environment and during mite-mite interactions. Furthermore, they may represent adapted structures of the mite with the purpose of housing specific beneficial microbes as found in other mite species. Tarsonemidae and *Trichouropoda* mites possess adaptations on the tegument to carry and protect fungal spores and bacteria (Moser 1985; Roets et al. 2011). These structures (sporothecae) are concave areas in which the legs are retracted (*Trichouropoda* spp.) (Roets et al. 2007). The specific areas of colonisation of T1 and T2 spores, on shield margins and coxal bases of phytoseiids might suggest that these structures represent rudimentary versions of the sporothecae.

Bacteria have been observed in the phytoseiid digestive tract in previous studies, but this work represents the first examination of their localization using FISH and advanced microscopy techniques. As with microbes on the body surfaces, bacteria within the (open) digestive tract showed specific areas of colonisation, particularly within the Malpighian tubules and anal atrium. It is possible that the phytoseiid digestive tract contains beneficial microbes that are necessary for digestion or improve nutrition as suggested in Astigmata and oribatids (Smrž and Trelová 1995; Hubert et al. 2015b). In Astigmata, FISH was also used to localize bacteria within the gut, suggesting a nutritional benefit (Hubert et al. 2011). Evidence from Erban et al. (2016) highlights the role of bacteria within the digestive tract of mites. Metabolism of food in these mites was associated with proteolytic activity in a *Bacillus cereus* strain associated with the dust mite *T. putrescentiae.* Addition of this bacterium to the diet of *T. putrescentiae* showed a significant increase in population growth compared to mites without the bacterium. This was probably due to the increased nutritional capacity of mites in association with *B. cereus* (Erban et al. 2016)*.* A similar function for the bacteria found within phytoseiids is posited here, furthermore as some species of phytoseiids are fed on factitious prey mites such as Astigmata, it is plausible that nutritional bacteria may be transmitted from prey to predator during feeding. Indeed, this phenomenon has been observed in the predatory mite *Cheyletus eruditus* (Schrank) (Acari: Cheyletidae) and its prey *Acarus siro* L. (Acari: Acaridae) (Hubert et al. 2016b).

This study highlights the phytoseiid body regions and specific structures with which microbes commonly associate. Recent studies have shown that phytoseiids are able to deposit entomopathogenic fungi to prey patches and that the anatomy of the mite is crucial in understanding the way it transmits these spores and how it protects itself from infection (Wu et al. 2016, 2018; Lin et al. 2019). Our study advances our knowledge of how microbes associate with their mite host and allows us to further understand how microbes and mites interact in situ.

In *Brevipalpus yothersi*, microfungi of the *Malasezzia* genus were found predominantly within the mycobiome (Rodrigues et al. 2019). Speculatively, T3 microbes found on the body of *T. swirskii* in the current study could be *Malassezia* given their size, shape and pattern of colonization on the epidermis of the mites observed (Dekker 2003). However, this requires further verification.

In *P. persimilis* the difference between batches was a significant factor describing the variation of microbe distribution and abundance. Both T1 (fungal conidia) and T2 (yeast-like) type microbes were affected by batch in *P. persimilis*, whereas only T3 microbes (microfungi-like) in *T. swirskii* were affected by this factor. This variation may be due to biotic or abiotic factors which change during rearing or transport. As the current study only analysed two time points, further studies should examine the Phytoseiidae microbiota consistently over long time periods to ensure seasonal changes are understood. Although only two batches were examined for each mite, long-term associations are present between Phytoseiidae mites and their microbiota as T1 and T2 microbes were found in both batches from different years. This could suggest a symbiotic relationship, but further study is needed to determine this.

The way in which microbes are transmitted helps to understand the nature of host-microbe interactions, providing information concerning the ecology of the microbe, whether they are reproductive parasites or occasional phoronts. In Phytoseiidae little is known of mechanisms that aid in the proliferation of microbial partners and pathogens throughout populations.

Female *N. cucumeris* were shown to have large amounts of T1 spores surrounding the genital opening (Fig. 3b), a structure located in the centre of the epigynal shield and used during oviposition. From this opening, the egg is deposited. The proximity of T1 microbes to this opening makes it likely that T1 microbes are transmitted from mother to egg during oviposition, thus presenting a method for vertical transmission of T1 microbes. This may represent a beneficial interaction or, alternatively T1 microbes are pathogenic and use this mechanism to infect offspring. Similar interpretations can be held for T2 microbes as these were frequently found on the epigynal shield close to the genital opening.

Further evidence suggests that microbes are transmitted horizontally during copulation. In the Phytoseiidae, mites mate via physical contact, venter to venter. Males deposit sperm into the female’s spermathecae, a structure located between the third and fourth coxal bases, close to the epigynal shield. This is facilitated via a specialised structure on the chelicerae called spermatodactyl (Amano and Chant 1978; Thomas and Zeh 1984). In these acts of copulation, it is possible that microbes are transferred from venter to venter (mite to mite) and from the mouthparts and legs of the male to the epigynal shield of the female. This may explain the high frequency of microbes associated with ventral body regions particularly the epigynal shield. A similar interaction has been suggested between male and female *Macrocheles* mites, where the male might be implicated in transferring fungi to the female during mating rituals and copulation (Perotti and Braig 2011).

Localization of bacteria (T4) in the anal atria of *T. swirskii*, *N. cucumeris* and *P. persimilis* (Fig. 7a–c), and particularly around the valves of the anal pore of *P. persimilis* (Fig. 7d) also suggests that bacteria are expelled from the internal organs of the host mite and may enter the environment. The microbes are likely to be encountered by conspecifics and may be dispersed throughout the population. This is facilitated when mites are reared in crowded environments or on factitious prey as are *N. cucumeris* and *T. swirskii* (Smytheman 2011; Mitdhassel et al. 2014)*.*

Furthermore, previous studies have suggested that grooming behaviours of phytoseiids are important in their defence against fungal pathogens and removal of fungal spores (Wekesa et al. 2007; Wu et al. 2014). The locations of microbes found in the current study could represent locations where the mites are not able to groom such as coxal bases, ventral areas and posterior sections of dorsal plates.

### Gut microbes and the role of the Malpighian tubules in phytoseiid microbiota

Work by Arutunyan (1985) mentions bacteria in the lumen of the alimentary tract of *P. persimilis* which entered the mite via food but did not cause adverse effects on the gut epithelium. It is likely that phytoseiids possess beneficial bacteria within their guts and that the entities discovered in the Malpighian tubules using FISH are beneficial ectosymbionts. Alternatively, they could represent an infective agent, as abnormalities in Malpighian tubules have previously been associated with symptoms of pathogens such as *Acaricomes phytoseiuli* (Bjørnson et al. 2000; Pukall et al. 2006; Schütte et al. 2008) and unidentified Rickettsia-like organisms (Hess and Hoy 1982).

Microscopic observations following FISH showed that phytoseiids contain bacteria within the lumen of their alimentary tract. This finding complements previous studies of the Acari and further supports the hypothesis that mites possess microbial communities within their internal organs (Hoy and Jeyaprakash 2005; Hubert et al. 2011; Pekas et al. 2017). These bacteria may aid the mites by maintaining the health of the digestive tract or providing other metabolic functions.

The Malpighian tubules (= post colonic diverticula), where bacteria were observed, open posteriorly into the anal atrium and consist of two parallel lobes which extend anteriorly terminating within the coxa of the first pair of legs (Krantz 2009). These structures are thought to store nitrogenous waste, reabsorb water and eventually expel waste into the anal atrium (Jakeman 1961; Evans 1992). The bacteria observed in the present study may play an important role in removal of waste and water re-absorption. Detailed studies by Coons and Axtell (1971) of the ‘excretory tubules’ (Malpighian tubules) of another member of the Mesostigmata, *Macrocheles muscaedomesticae* (Scopoli), describe excretory granules in the lumen of the Malpighian tubules that resemble bacteria. The bacteria observed in the present study may have a role in forming or metabolising these excretory crystals. It is also possible that the Malpighian tubules and anal atria in phytoseiids have a role in removing from the digestive tract, excess bacteria that cannot otherwise be metabolised or excreted. Further examples of bacteria within the Malpighian tubules of the honeybee parasite *Varroa destructor* Anderson & Trueman (Liu and Ritter 1988; Hubert et al. 2015a) suggest that these structures play an important and uncovered role regarding microbe and mite interactions. This evidence of a bacterial related function of the Malpighian tubules also supports the recent suggestions of microbe mediated compartmentalisation within the gut of mites as posited for *Carpoglyphus lactis* L. (Acari: Astigmata) (Hubert et al. 2015b).

## Conclusion

The external microbiota of phytoseiids is a dynamic, yet unknown, aspect of their biology. Four morphologically distinct types of microorganisms were observed during this study including yeast-like organisms, possible fungal conidia and spores and rod-shaped bacteria. They colonised specific areas of the body and some were present throughout batches suggesting a lasting or long-term relationship between microbe and mite host. This study has introduced unknown partners that appear to live in association with phytoseiids. In advance of further research which focuses on characterizing the microbiota of phytoseiids it is critical to understand how both partners interact with each other in-situ. Additional research on the identity of these fungus-like and bacterial symbionts will shed light on the nature of these interactions. Furthermore, this study shows that basic microscopy techniques are still a useful tool in exploring the microbial ecology of micro-invertebrates. However, coupled with molecular techniques these associations can be explored even further by identifying microbial partners. Generally, studies on the microbiota of invertebrates and vertebrates continue to reinforce their importance, emphasising the need for further research in this subject within the Acari, especially of economically important taxa.
